# The energy sensor OsSnRK1a confers broad-spectrum disease resistance in rice

**DOI:** 10.1038/s41598-018-22101-6

**Published:** 2018-03-01

**Authors:** Osvaldo Filipe, David De Vleesschauwer, Ashley Haeck, Kristof Demeestere, Monica Höfte

**Affiliations:** 1 Laboratory of Phytopathology, Department Plants and Crops, Faculty of Bioscience Engineering, Ghent University, Coupure Links 653, 9000 Ghent, Belgium; 2 Research Group EnVOC, Department of Green Chemistry and Technology, Faculty of Bioscience Engineering, Ghent University, Coupure Links 653, 9000 Ghent, Belgium; 3 Present Address: Bayer CropScience NV, Technologiepark 38, 9051 Zwijnaarde, Belgium

## Abstract

Sucrose non-fermenting-1-related protein kinase-1 (SnRK1) belongs to a family of evolutionary conserved kinases with orthologs in all eukaryotes, ranging from yeasts (SnF1) to mammals (AMP-Activated kinase). These kinases sense energy deficits caused by nutrient limitation or stress and coordinate the required adaptations to maintain energy homeostasis and survival. In plants, SnRK1 is a global regulator of plant metabolism and is also involved in abiotic stress responses. Its role in the response to biotic stress, however, is only starting to be uncovered. Here we studied the effect of altered SnRK1a expression on growth and plant defense in rice. *OsSnRK1a* overexpression interfered with normal growth and development and increased resistance against both (hemi)biotrophic and necrotrophic pathogens, while *OsSnRK1a* silencing in RNAi lines increased susceptibility. *OsSnRK1a* overexpression positively affected the salicylic acid pathway and boosted the jasmonate-mediated defense response after inoculation with the blast fungus *Pyricularia oryzae*. Together these findings strongly suggest OsSnRK1a to be involved in plant basal immunity and favor a model whereby OsSnRK1a acts as a master switch that regulates growth-immunity trade-offs.

## Introduction

Rice (*Oryza sativa*) is a staple food for more than half of the world’s population and a scientific model for cereal crops and other monocots, providing about 20 percent of direct human calorie intake world-wide. Its consumption exceeds 100 kilograms per capita annually in many Asian countries making rice one of the most important cereals on earth. However, with a fast growing world population, rice production can barely keep pace with consumption and should increase by more than 40 percent above today’s level, if the world’s food supplies are to be secured until the year 2030^1^. About half of the increased production that will be needed in the future must come from optimized varieties, and the rest from better growing techniques, as the area of land available to rice growers cannot be enlarged^1,2^. Stress resistance, better nutrient uptake and optimized grain quality are also important. Diseases caused by bacteria, viruses, or fungi greatly reduce rice yield and planting a resistant variety is the simplest and, often, the most cost effective management for diseases. Amongst the most serious and widespread diseases of cultivated rice, blast disease, caused by the fungus *Pyricularia oryzae* (syn. *Magnaporthe oryzae)* and bacterial leaf blight disease caused by *Xanthomonas oryzae pv*. *oryzae* (*Xoo*), continuously threaten rice production worldwide^3–6^. Although increasing host plant resistance by introducing resistance genes (R genes) is an ecologically sound way to manage crop diseases, race specificity and the potential risks of resistance breakdown have limited their usefulness. Additionally, defense responses usually have fitness costs, reflecting the trade-off between disease resistance and plant growth^7^. In recent years a plethora of studies in plant-pathogen interaction have provided mechanistic insights into the plant response to potential pathogens and identified several signaling molecules, including sugars, hormones, protein kinases and transcription factors that mediate the translation of pathogen perception into defense responses^8–17^. Crosstalk between the different signaling pathways suggests an antagonism between growth and defense, involving resource reallocation from growth to defense, which is of crucial importance for plant survival. Therefore, understanding the mechanisms that regulate this innate trade-off is critical to prevent yield loss and control plant diseases.

In eukaryotic organisms, ‘fuel gauges’ such as sucrose non-fermenting1 (SNF1) in yeast, and AMP-activated kinase (AMPK) in mammals, are central to the control of energy homeostasis. These evolutionarily conserved heterotrimeric serine/threonine kinases act as energy sensors, triggering the activation of catabolic processes and the repression of energy-consuming anabolic processes and growth. In plants, SNF1-related kinases (SnRKs) emerge as promising candidates to balance growth and defense^18–21^. Unlike yeast and mammals, plants carry a large family of SnRKs, which can be subdivided into three subfamilies (SnRK1, SnRK2 and SnRK3), of which SnRK1 is closest related to yeast and mammalian counterparts, enabling plants to link metabolic and stress signaling differently from other organisms^22–24^. Increasing evidence, mostly from *Arabidopsis thaliana* studies, indicates that SnRKs play an important role in plant biotic interactions. SnRK1 overexpressing plants display increased resistance to geminiviruses, while SnRK1 silenced plants are more susceptible than wild-type plants^25,26^. In contrast, SnRK2 and SnRK3 subfamilies play specific roles in responses to abiotic stress conferring tolerance to drought, salt, and low temperature and are important in abscisic acid (ABA) signaling in different crops^27–31^. These studies implicate SnRKs in the regulation of metabolic homeostasis and hormonal metabolic regulation of growth and development. Despite the relative wealth of information available in yeast and mammals, it remains unclear how plant SnRK1 senses and transduces metabolite, energy and defense signals in order to influence disease outcomes^32–36^.

Rice has three SnRK1 α-subunit genes which can be subdivided into two subgroups, SnRK1a (OSK1) and SnRK1b (OSK24 and OSK35), which based on both amino acid sequence similarities and expression patterns may play distinct roles^37^. SnRK1a functions in the sugar signaling cascade, upstream of a MYB gene, *MYBS1*, and an α-amylase gene, *Amy3*, playing a key role in regulating seed germination and seedling growth^38^. OSK24 may have a specific role in regulating starch accumulation^39^, while OSK35 positively regulates defense against *P*. *oryzae* and *Xoo*^19^.

Here we demonstrate through a multidisciplinary approach using molecular, genetic, biochemical and pathological analyses, that in rice, in addition to regulating growth and development, the SnRK1a gene confers broad and durable disease resistance, while potentiating the responses of the classic defense hormones salicylic acid (SA) and jasmonate (JA).

## Results

### OsSnRK1a shares common architecture with SNF1/AMPK/SnRK1

**Figure 1:**
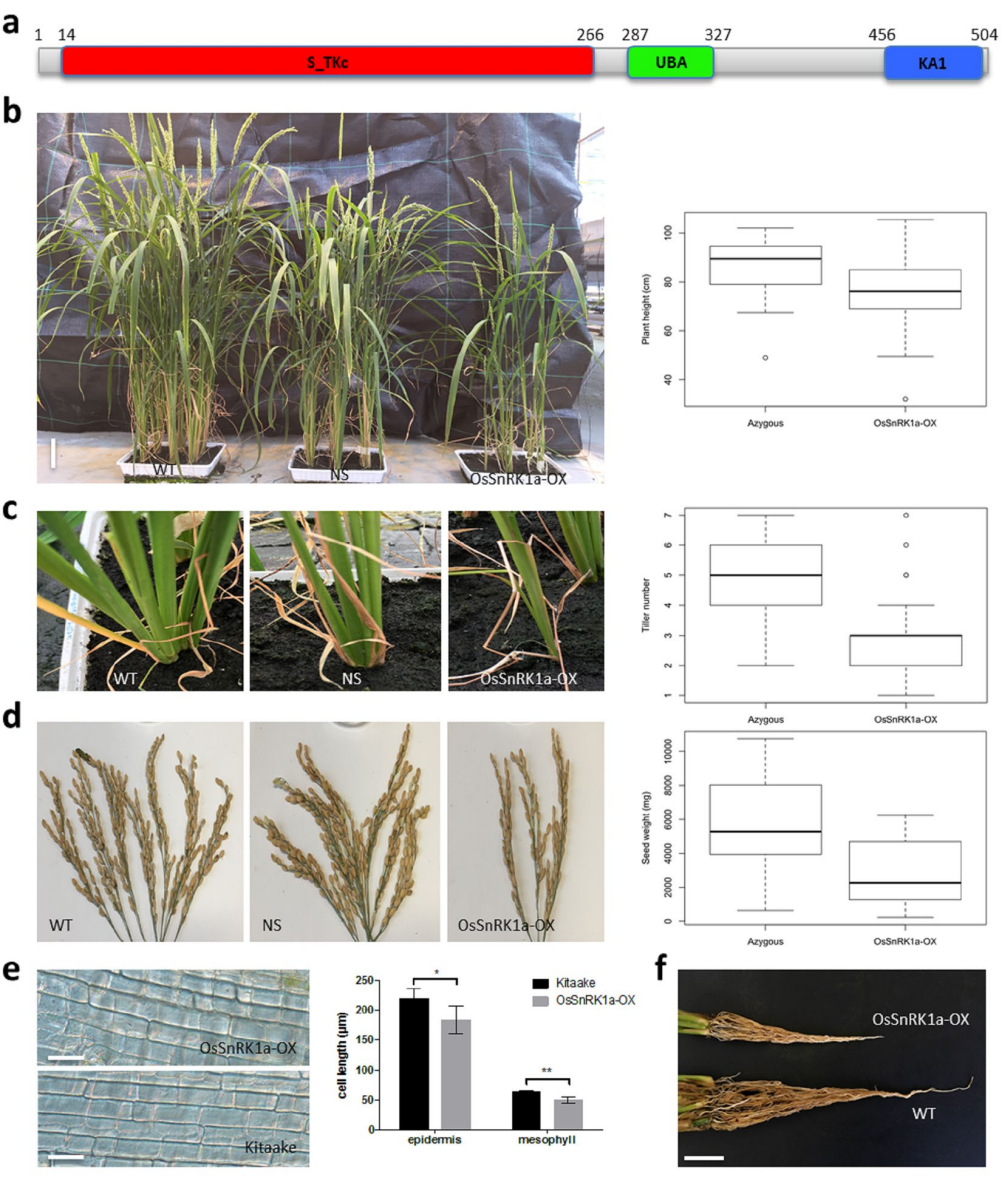
*OsSnRK1a* overexpression negatively affects plant growth and development. (**a**) Diagrammatic representation of the rice Sucrose Non-fermenting-1 related kinase 1 A (OsSnRK1a). Protein motif and domains of OsSnRK1a were identified using the Simple Architecture Research Tool (SMART) (http://smart.embl-heidelberg.de) and NCBI-CDD. (http://www.ncbi.nlm.nih.gov/Structure/cdd/wrpsb.cgi). S_TKc, Serine/Threonine protein kinase catalytic domain; UBA, ubiquitin-associated domain; KA1, kinase-associated 1 motif. (**b**) to (**f**) Phenotypes of T1 *OsSnRK1a* overexpressing transgenic rice plants. Azygous controls = null segregants (NS, progeny of lines *OsSnRK1a-*OX 13–2, 17–4) and Kitaake wild type (WT). *OsSnRK1a-*OX = progeny of *OsSnRK1a*-overexpressing lines 5-1, 5-3, 9-5, 10-5, 14-2, and 14-8. When 12 weeks old, plants were scored for several growth parameters. Overexpression of *OsSnRK1a* inhibited (**b**) plant growth, (**c**) tiller number, (**d**) seed yield, (**e**) epidermal and mesophylic cell elongation, and (**f**) root growth. Box plots in (**b**) to (**d**) show box and whiskers (Min to Max), open circles represent outliers outside the range of the whiskers. Welsh’s t-test for (**b**), (**c**) and (**d**) revealed significant results with p = 0.001621, p = 0.0005032, and p = 0.001197, respectively. Pictures are representative of the lines in bold. Bars in (**b**), (**e**) and (**f**) are 7 cm, 50 μm and 3 cm respectively. Pictures in (**d**) show seed yield of a single representative plant at week 13. Asterisks in (**e**) represent significant differences compared to Kitaake plants (Mann-Whitney test, ns = p > 0.05, *p ≤ 0.05; **p ≤ 0.01; ***p ≤ 0.001).

A full-length *OsSnRK1a* cDNA, which encodes the catalytic α-subunit of the SnRK1 complex, was isolated from rice leaf tissue using PCR. The 1518 bp ORF encodes the OsSnRK1a protein with 505 AA residues, a predicted molecular weight of 57.6 kDa, and an isoelectrical point (pI) of 8.02, respectively. As reported previously^40^, using the Simple Architecture Research Tool (SMART) and InterPro we found that, similar to *Arabidopsis*^41^, the amino acid sequence of OsSnRK1a comprises an amino (N)-terminal Serine/Threonine kinase catalytic domain (S_TKc) followed by an Ubiquitin-associated domain (UBA) and a kinase-associated 1 (KA1) domain at its carboxyl (C)-terminal (Fig. 1a). Detailed amino acid sequence and protein information can be found in Supplementary Fig. S1.

### OsSnRK1a negatively regulates growth and development in rice

To better understand the role of OsSnRK1a in plant growth and development we cloned the full-length *OsSnRK1a* gene by PCR and generated transgenic rice lines by expressing the cloned gene under control of a constitutive (maize ubiquitin) promoter. Six independent T_0_ lines that contained the hygromycin construct and overexpressed *OsSnRK1a* (*OsSnRK1a*-OX) lines were obtained. Out of the progeny of 9 T1 lines, 35 lines were randomly selected for further genotyping and characterization (Supplementary Table S2). In addition we made use of *OsSnRK1a-*RNAi lines that had been generated before in Kitaake containing the pattern recognition receptor (PRR) Xa21 (Kit-Xa21)^18^. Xa21 confers resistance to various *Xoo* strains, including PXO99.

Consistent with previous reports^20,38,42^, where SnRK1 has been linked to sugar signaling and plant development, we found that increased levels of *OsSnRK1a* interfered with normal growth and development. Compared to wild-type Kitaake and azygous control plants, *OsSnRK1a-*OX plants showed diverse phenotypes including shorter shoots, decreased tillering and biomass accumulation, reduced root growth and decreased seed yield (Fig. 1b–d,f and Table S2). In addition, *OsSnRK1a* overexpression delayed flowering and inhibited epidermal and mesophylic cell elongation compared to wild-type Kitaake and azygous controls (Fig. 1e and Supplementary Fig. S2). In contrast, flowering seems to be promoted in the *OsSnRK1a-*RNAi lines (Supplementary Fig. S2). To validate earlier work showing that increased SnRK1 signaling extends lifespan in a number of model organisms^20,43^, we performed a detached leaf assay where *OsSnRK1a-*OX lines were found to display delayed senescence-associated yellowing and lower percentage of senescent leaves compared to wild-type Kitaake and null segregants (Supplementary Fig. S3). Moreover, these phenotypes correlated with the presence of the transgene as verified by polymerase chain reaction (PCR) genotyping (Supplementary Table S2). Together these results corroborate previous findings^44^ demonstrating a role for SnRK1 in the regulation of plant growth and development.

### OsSnRK1a confers broad-spectrum resistance

**Figure 2:**
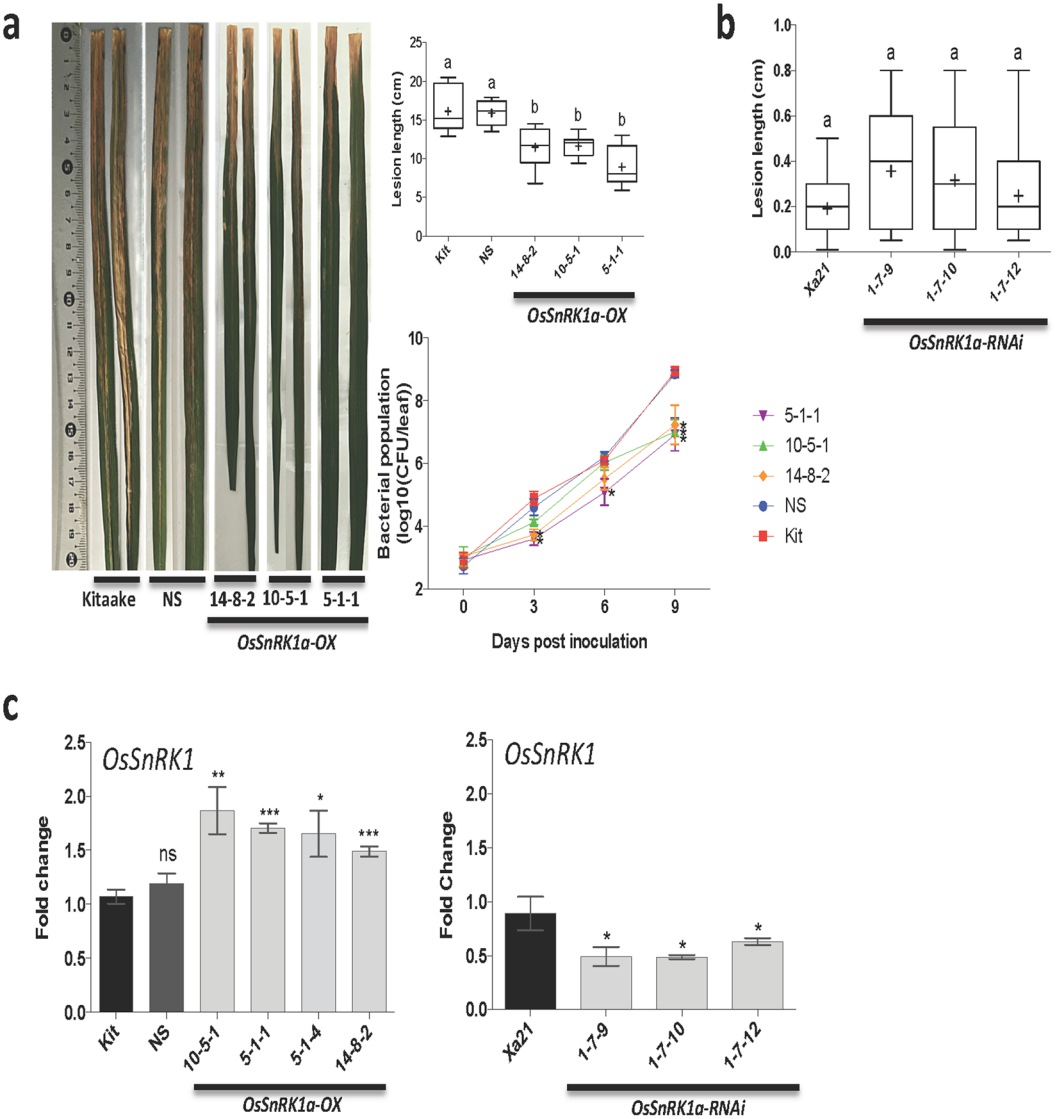
*OsSnRK1a* overexpression positively correlates with resistance to bacterial blight caused by *Xanthomonas oryzae pv*. oryzae (*Xoo*). (**a**) Fully developed fifth leaf of 6-week-old progeny of overexpressing *OsSnRK1a* (lines 5-1-1, 10-5-1 and 14-8-2), wild-type Kitaake and null segregating (NS, line 17-4-1) plants, grown in growth-chamber were inoculated and disease was evaluated by measuring the length of the water-soaked leaf blight lesion and assessing bacterial growth in planta. Box and whiskers (Min to Max) with the mean shown as a ‘+’ sign. Different letters indicate statistically significant differences (Tukey, n ≥ 12, α = 0.05). Bacterial population data are means ± SE of three leaves from sibling progeny of the plant indicated, each with four technical replicates. Within each time point, asterisks indicate statistically significant differences compared to control treatments (LSD, n = 4, α = 0.05). (**b**) *OsSnRK1a* down-regulation failed to induce susceptibility towards Xoo in the Xa21 genetic background. (**c**) RT-qPCR results show upregulation of *OsSnRK1*a transcript in progeny from segregating (*OsSnRK1-*OX) lines, and downregulation in the Xa21 genetic background mutants (*OsSnRK1a*-RNAi). Lines 5-1-1 and 5-1-4 are T_1_ sibling plants. Data are means ± SD of three technical and three biological replicates, with each biological replicate representing a pooled sample from three individual plants. Asterisks represent significant difference compared to Kitaake or Xa21 plants (t-test, ns = p > 0.05, *p ≤ 0.05; **p ≤ 0.01; ***p ≤ 0.001).

To investigate whether OsSnRK1a plays a role in plant immunity and whether the role of OsSnRK1a depends on the lifestyle and/or infection strategy of the invading pathogen, we challenged our overexpression lines, and RNAi^18^ lines, with various rice pathogens, including *Xoo* PXO99 and *P*. *oryzae* VT5M1 (hemibiotrophs) and *C*. *miyabeanus Cm988* and *R*. *solani AG1-1A* strain 16 (necrotrophs). As control for *OsSnRK1a-OX* we used the wild-type background cultivar Kitaake along with a randomly selected control azygous line (progeny of line *OsSnRK1-*OX 17-4; further described as null segregant). For the *OsSnRK1a*-RNAi lines Kit-Xa21 was used as control. First, eight-week-old progeny from 9 T1 *OsSnRK1a-*OX lines was examined for co-segregation of genotype with the phenotype by PCR analysis and measurements of the length of the *Xoo*-induced leaf blight lesions (Supplementary Table S2). Segregation of the construct correlated well with resistance against *Xoo* as plants carrying the transgene displayed significantly enhanced resistance to the virulent *Xoo* strain PXO99, with average lesion lengths of 15.6 cm compared to 20.3 cm on azygous control lines. To further extend these findings, six-week-old progeny of three independent T2 *OsSnRK1a-*OX lines was challenged with *Xoo* and disease was evaluated by measuring the length of the water-soaked leaf blight lesion and bacterial titers at different times. We found that bacterial growth correlated well with lesion length developments (Fig. 2a). Starting at 3 dpi, the growth of *Xoo* was significantly suppressed in the *OsSnRK1a-*OX lines 14-8-2 and 5-1-1 compared to wild-type Kitaake and null segregants. At 9 dpi, PXO99 titers reached less than 1 × 10^8^ cfu/leaf in all three *OsSnRK1a-*OX lines, a more than 10-fold decrease compared to wild-type Kitaake and null segregants. Moreover, RT-qPCR analysis revealed that the level of endogenous *OsSnRK1a* was significantly higher in our overexpression lines compared to wild-type Kitaake and null segregants and lower in *OsSnRK1a* silenced plants compared to Kit-Xa21 (Fig. 2c). However, the *OsSnRK1a*-RNAi lines were as resistant to *Xoo* PXO99 as the Kit-Xa21 control plants (Fig. 2b).

**Figure 3:**
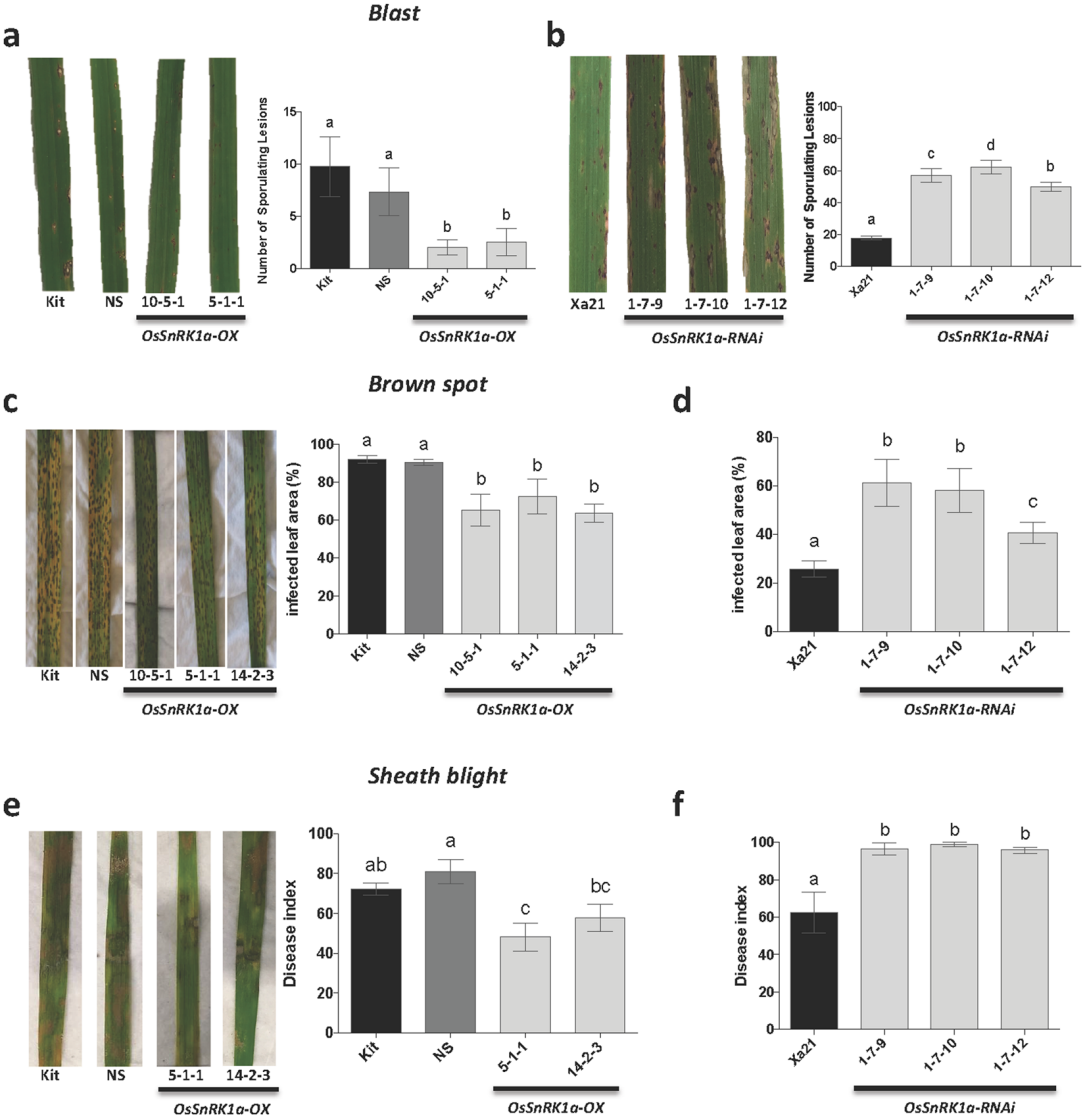
*OsSnRK1a* positively affect resistance against fungal pathogens *in rice*. (**a**) and (**b**) *OsSnRK1a* upregulation and downregulation mount resistance and susceptibility, respectively, against the rice blast pathogen *Magnaporthe oryzae*. Five-leaf stage rice plants were challenged by spraying a spore suspension of a moderately virulent *M*. *oryzae* strain, VT5M1 (4.5 × 10^5^ spores.mL^−1^ in a; 1.0 × 10^5^ spores.mL^−1^ in b), and only their second youngest leaf was scored. Pictures depicting representative symptoms were taken and sporulating lesions were counted 7 d after inoculation. Data are means ± SD and different letters indicate statistically significant differences (Tukey, n ≥ 3, α = 0.05). (**c**) *OsSnRK1a* overexpression decreases susceptibility towards brown spot disease caused *Cochliobolus miyabeanus*. T3 lines overexpressing *OsSnRK1a* (10-5-1, 5-1-1 and 14-2-3) were challenged with *C*. *miyabeanus* strain Cm988 (0.75 × 10^4^ spores/mL in 0.5% gelatin). (**e**) *OsSnRK1a* overexpression also protects against infection by the sheath blight pathogen *Rhizoctonia solani*, while *OsSnRK1a* down-regulation in Xa21 genetic background increases susceptibility towards the necrotrophic rice pathogens *C*. *miyabeanus* (**d**) and *R*. *solani* (**f**). Leaves showing representative disease symptoms (c and e) were photographed 4 and 5 days post inoculation, respectively. Detached leaf essays (**c**–**f**) were repeated at least twice with similar results and data from only one representative experiment is shown. Data are means ± SD and different letters indicate statistically significant differences (Tukey, n ≥ 18, α = 0.05).

To determine if altered *OsSnRK1a* expression can also affect rice immune response to a fungal pathogen, we next evaluated resistance levels of *OsSnRK1a-*OX and *OsSnRK1a*-RNAi lines to the moderately virulent *P*. *oryzae* strain VT5M1. Plants overexpressing *OsSnRK1a* displayed a decrease in number of susceptible-type sporulating lesions. Moreover, *OsSnRK1a-*OX plants exhibited mainly nonsporulating necrotic spots, characteristic of resistance phenotypes (Fig. 3a). Downregulation of *OsSnRK1a* in the Xa21 genetic background led to increased susceptibility to the rice blast fungus compared to controls (Fig. 3b).

To further examine whether OsSnRK1a also affects resistance to necrotrophic pathogens, additional assays were performed with the brown spot fungus *Cochliobolus miyabeanus* and the sheath blight pathogen *Rhizoctonia solani*. In contrast to biotrophs, which infect and feed on living cells, necrotrophs kill host tissue as they colonize and thrive on the contents of dead or dying cells^14^. Three independent transgenic lines overexpressing *OsSnRK1a* and *OsSnRK1a*-RNAi lines were challenged with *C*. *miyabeanus* strain Cm988 and *R*. *solani* AG1-1A strain 16. Strikingly and in keeping with the results obtained with *Xoo* and *P*. *oryzae*, inoculation with *C*. *miyabeanus* revealed that OsSnRK1a positively affects resistance against brown spot disease. *OsSnRK1a* overexpression leads to enhanced resistance, whereas *OsSnRK1a* silencing increases susceptibility to Cm988 strain compared to Kitaake and Kit-Xa21 respectively. Similarly, *OsSnRK1a-*OX plants showed decreased susceptibility towards *R*. *solani*, while *OsSnRK1a* silencing increased susceptibility (Fig. 3c–f).

### OsSnRK1a potentiates the action of the archetypal defense hormones salicylic acid and jasmonate

**Figure 4:**
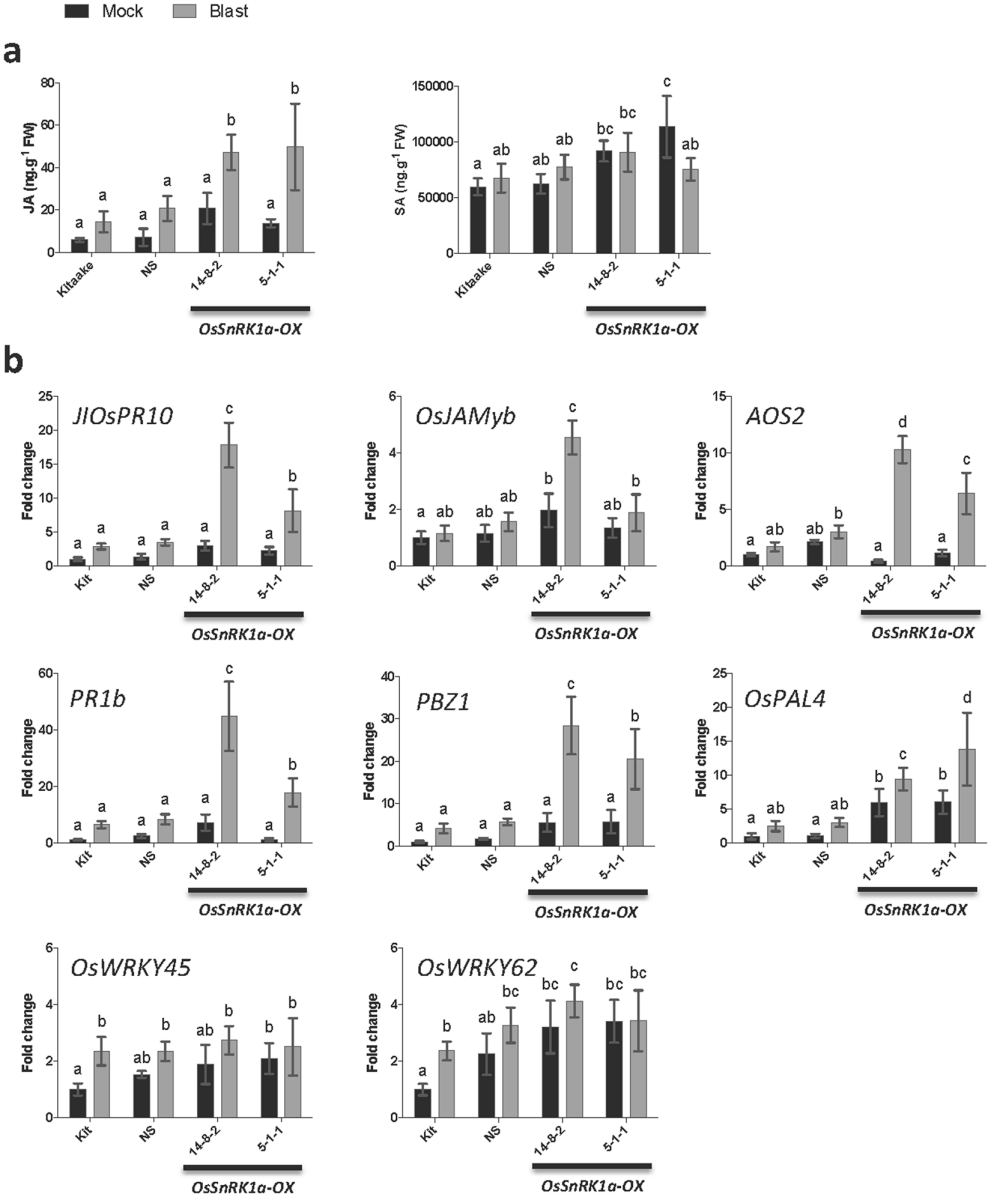
*OsSnRK1a* orchestrates SA and JA signaling and upregulates defense genes during blast infection. Five-leaf stage rice plants were challenged by spraying a spore suspension of a moderately virulent *M*. *oryzae* strain, VT5M1 (4.5 × 10^5^ spores.mL^−1^), and only the second youngest leaf of each plant was sampled. Samples were taken at 4 dpi. (**a**) Quantification of endogenous JA and SA levels. Overexpression of *OsSnRK1a* increased endogenous JA levels, especially during pathogen attack, while *OsSnRK1a* upregulation did not significantly alter SA contents compared to controls during blast infection. Data are means ± SD from at least three biological replicates each representing a pooled sample from three individual plants. Different letters indicate statistically significant differences (Tukey, n ≥ 3, α = 0.05). (**b**) Effect of *M*. *oryza*e inoculation on SA- and JA-responsive genes in wild-type Kitaake, null segregant (NS, line 17-4-1) and T3 *OsSnRK1a-*OX (lines 5-1-1 and 14-8-2) plants. Transcript levels were normalized using actin as an internal reference and expressed relative to the normalized expression levels in mock-inoculated Kitaake plants. Data are means ± SD of two technical and three biological replicates, each representing a pooled sample from three individual plants. Different letters indicate statistically significant differences (Tukey, α = 0.05).

In an attempt to unravel the mechanisms by which OsSnRK1a modulates plant responses to pathogen attack, we looked at the effect of *OsSnRK1a* overexpression or downregulation on the classic defense hormone pathways SA and JA using the rice-blast pathosystem. We measured SA and JA levels in *OsSnRK1a*-OX and *OsSnRK1a*-RNAi lines and their corresponding control plants at 4 days after *P*. *oryzae* inoculation and mock treatment. Our results showed that *OsSnRK1a* overexpression boosts JA production but not SA production after blast infection (Fig. 4a). No differences were found in SA and JA levels during blast infection between the *OsSnRK1a*-RNAi lines and their control Kit-Xa21 (data not shown).

To get more insight into OsSnRK1a-SA/JA crosstalk, we tested the effect of *OsSnRK1a* overexpression on the expression of JA- and SA-responsive genes and looked also at its effect on PR genes expression 4 days after *P*. *oryzae* inoculation. Consistent with OsSnRK1a enhancing JA signaling, *OsSnRK1a* overexpression boosted expression of the JA-marker genes *JIOsPR10* and *OsJAMyb* and JA biosynthetic gene AOS2 after *P*. *oryzae* inoculation. *OsSnRK1a* overexpression also primed the expression of SA- and JA-responsive pathogenesis-related genes *PR1b* and *OsPR10a/PBZ1* in plants inoculated with *P*. *oryzae* and increased expression of the defense related *OsPAL4* gene during normal and inoculated conditions (Fig. 4b). OsSnRK1a seems to positively affect the expression of the SA-inducible transcription factors OsWRKY45 and OsWRKY62 under normal conditions especially in older plant (10 dpi, Supplementary Fig. S4), but no further increase in expression was observed in *OsSnRK1a*-OX plants inoculated with *P*. *oryzae* (Fig. 4b and Supplementary Fig. S4).

## Discussion

In recent years, several studies have suggested an important role for SnRK1-mediated signaling in plant biotic interactions. However, the exact mechanisms involved in the regulation of this evolutionarily conserved kinase remain largely elusive and are likely diverse and very complex. Moreover, an increasing body of evidence supports metabolic and hormonal regulation of SnRK1 activity, pointing to the fact that this kinase might function as a master switch through which hormonal and energetic cues may coordinate growth-defense tradeoffs^40,45,46^. These findings suggest that SnRK1 is central for plant metabolic regulation by translating internal and external cues within and across cells, thereby providing plants with a considerable degree of plasticity to grow and survive under challenging environments.

In rice, overexpression of *OsSnRK1a* leads to reduced shoot and root growth, and delays flowering and senescence (Fig. 1 and Supplementary Figs S2–S3). This confirms results obtained in *A*. *thaliana*, where expression of inactive SnRK1 leads to transgenic seedlings with longer root lengths^20^ and overexpression of the catalytic subunit of SnRK1 delays flowering and senescence^44,45,47,48^. Corroborating our findings showing that SnRK1 plays a role in senescence, recently SnRK1 was reported to delay leaf senescence by direct interaction and repression of the transcription factor EIN3 (ethylene-insensitive3) in Arabidopsis^49^.

Opposite to current beliefs, where SnRK1 is expected to be mostly relevant for interactions with biotrophic pathogens^46^, we demonstrate that OsSnRK1a positive effect on plant resistance is independent of pathogen lifestyles and/or infection strategies, since *OsSnRK1a* overexpression increases resistance to both (hemi)biotrophic (*Xoo* and *P*. *oryzae*,) and necrotrophic (*C*. *miyabeanus* and *R*. *solani*) pathogens, while downregulation leads to susceptibility (Figs 2–3). It has been shown before that silencing of the rice catalytic α-subunit OsSnRK1a abolishes Xa21-mediated resistance to *Xoo*^18^, suggesting a link between OsSnRK1a and Xa21-mediated responses. However, under our experimental conditions Xa21- and OsSnRK1a-triggered immunity appear to function independently from each other during pathogen attack, since *OsSnRK1a-*RNAi lines generated in the Kit-Xa21 background were as resistant to *Xoo* as the corresponding Kit-Xa21 control plants (Fig. 2), while they are clearly more susceptible to other pathogens (Fig. 3). This apparent contradiction might be due to differences in experimental conditions such as plant age or environment and needs further investigation.

It is worth noting that SnRK1 has also been associated with plant hormone signaling, as ectopic expression of SnRK1 renders Arabidopsis hypersensitive to ABA^50^, while SnRK1 down-regulation in pea links SnRK1 to auxin and cytokinin signaling pathways^44,51^. In keeping with these studies, Eichmann & Schäfer^52^ proposed a model explaining growth-defense trade-offs via cell cycle regulations, in which hormones are crucial in redirection of cell cycle function from growth into immunity during biotic stress^52^. However, to date the effect of SnRK1 activity on the major plant defense hormones SA and JA remained to be investigated^46^. SA and JA play a well-studied role in establishing immunity against various types of pathogens^13,14,53,54^. We propose that OsSnRK1a-mediated immunity is due to its ability to crosstalk with different hormones and at least in part by potentiating the action of SA and JA. We found that expression of the SA-responsive transcription factors OsWRKY45 and OsWRKY62 is positively influenced by *OsSnRK1a* overexpression, which becomes more clear in older plants (Fig. 4 and Supplementary Fig. S4). Blast infection did not lead to a further increase in the expression of these SA-response WRKY factors (Fig. 4b and Fig. S4). It has been shown that *OsWRKY45* overexpression leads to a strong resistance against *P*. *oryzae* and *Xoo*^55^, while *OsWKRY62* knock down rice was significantly more susceptible to both pathogens. OsWRKY62 and OsWRKY45 can form a heterodimer that acts as a strong activator of plant defense^56^. Endogenous SA levels increased in one of the two *OsSnRK1a-*OX lines. However, no differences in SA levels were observed among the different lines upon *P*. *oryzae* inoculation. The latter confirms earlier studies showing that distinct from Arabidopsis, endogenous SA levels in rice leaves are already very high and do not change significantly upon fungal and bacterial pathogens attack (Fig. 4a) (for review see^13,14^). These data suggest that *OsSnRK1a* overexpression positively affects the SA pathway in the absence of pathogens. In contrast, effects of *OsSnRK1a* overexpression on JA levels and the expression of JA-responsive genes (Fig. 4b) are only seen upon *P*. *oryzae* inoculation, indicating a priming effect on the JA pathway (Fig. 4a). Defense priming is defined as the sensitization of a plant for enhanced defense, which results in faster and more robust activation of defense responses upon challenge^57^. The increase of endogenous JA and stronger induction of SA- and JA-responsive genes by *OsSnRK1a* overexpression appear to be translated into the enhanced resistance of transgenic rice to the blast fungus (Figs 3 and 4). Transgenic rice plants overexpressing *OsAOS2*, encoding a key enzyme in JA biosynthesis, display increased endogenous JA levels, enhanced activation of PR genes and disease resistance against rice blast^58^. Finally, induced expression of the *OsPAL4* gene was already observed in *OsSnRK1a*-OX lines in the absence of pathogens, and expression was further induced upon *P*. *oryzae* inoculation (Fig. 4). PAL is a key enzyme in the phenylpropanoid pathway. Products of this pathway contribute to disease resistance and include lignin, phenolics and flavonoids. Moreover, PAL is also a key enzyme in SA biosynthesis. OsPAL4 is associated with broad spectrum disease resistance and contributes to QTL-mediated resistance to bacterial blight and sheath blight. Moreover, a heterozygous *ospal4* mutant line showed increased susceptibility to bacterial blight, sheath blight and rice blast^59^. SA and JA signaling pathways may feed into a common defense system that is not only effective against *P*. *oryzae*, but also against *Xoo* and *R*. *solani*^4^. In this context, it was demonstrated that overexpression of WRKY30, a transcription factor that is involved in both SA and JA pathways, makes rice transformants resistant to both (hemi)biotrophic pathogens (*P*. *oryzae* and *Xoo*) and *R*. *solani*, a necrotrophic pathogen^60,61^. However, possible effects on the JA and SA pathway cannot explain the observed increase in resistance against the necrotrophic pathogen *C*. *miyabeanus*^10^. This pathogen is known to hijack the ethylene pathway to cause disease in rice^62^. We hypothesize that the increased brown spot resistance in *OsSnRK1a-*OX lines might be due to a delay in senescense and a possible suppression of the ET response^49^. However, in rice the role of OsSnRK1a on the ethylene pathway remains to be investigated.

SnRK1 control of growth and development may be particularly critical in the context of costs and trade-offs associated with disease resistance. In mammals and yeast SnRK1 acts antagonistically with the target of rapamycin (TOR)^63^. TOR is an evolutionary conserved Ser/Thr kinase, which acts as a master regulator that promotes growth and development in response to environmental cues^64^. In plants TOR is also heavily intertwined with hormone signaling pathways^45,65,66^. We have recently shown that rice lines overexpressing TOR become more susceptible to *Xoo*, *C*. *miyabeanus* and *R*. *solani* while downregulation of TOR increased resistance to these pathogens. TOR antagonizes plant defenses by interfering with the classic defense hormones SA and JA^67^. Moreover, in accordance with our results where *OsSnRK1a* overexpression induced JA levels in rice, Song *et al*.^68^ showed that TOR inhibition by an ATP-competitive TOR specific inhibitor, AZD8055, increases JA levels in cotton and Arabidopsis. Therefore, it is tempting to suggest that the two hormones, SA and JA, signal through the SnRK1-TOR regulatory module, linking growth to defense^21,32,45,69^.

Our study not only shows for the first time the function of OsSnRK1a in the biosynthesis of JA in monocotyledonous species, but also demonstrates the importance of OsSnRK1a action in mediating rice PR gene expression and broad-spectrum disease resistance. Fine-tuning of SnRK1 activity or its target processes may be of great value in the near future in preventing yield loss and treatment of plant diseases. SnRK1 could be a promising target for direct or indirect modification and selection for broad-spectrum and sustainable resistance. For instance, SnRK1 agonists may be used as resistance inducers to prevent yield loss due to pathogens and circumvent fitness costs seen in SnRK1 overexpression lines. Alternatively, transgenic overexpressing SnRK1 lines under a pathogen responsive promoter hold great promise for improving crop quality and productivity. Therefore, understanding the mechanisms that regulate SnRK1-mediated defenses will be instrumental in the prevention of yield loss and treatment of plant diseases.

## Materials and Methods

### Plant materials and growth conditions

The *OsSnRK1a* overexpressing, *OsSnRK1a-*OX, and silencing, *OsSnRK1a*-RNAi^18^ rice (*Oryza sativa* L.) lines, together with their respective wild-types Kitaake and Kit-Xa21, were used in this study. All rice seeds were routinely dehulled and surface sterilized by agitation in 2% sodium hypochlorite for 25 min, rinsed three times with sterile demineralized water and germinated for 4 days at 28 °C on sterilized wet filter paper. Germinated seedlings were placed in autoclaved commercial potting soil (Structural; Snebbout, Kaprijke, Belgium) and grown under greenhouse conditions (28 ± 2 °C, 12 h photoperiod) or under growth chamber conditions (28 °C day/24 °C night, 12 h photoperiod, and 70% relative humidity). Plants were fertilized weekly until flowering with a solution containing 0.2% iron sulfate and 0.1% ammonium sulfate.

### Gene cloning and plasmid constructs

The coding sequence of *OsSnRK1a* (LOC_Os05g45420) was amplified using cDNA isolated from 2-week-old Kitaake plants as PCR template. Total RNA was isolated using Trizol (Invitrogen) and treated with Turbo DNA-free DNAse (Ambion) following the manufacturer’s protocol. Two µg of total RNA was used for cDNA synthesis using Superscript II Reverse Transcriptase (Thermo Fisher Scientific) and gene-specific reverse transcriptase (RT) primers (Supplementary Table S1). PCR products of the correct size were gel purified and cloned into pENTR/D-TOPO vector (Invitrogen). We verified the DNA sequence of the inserts by standard Sanger sequencing and recombined the full length *OsSnRK1a* coding sequence into the Gateway-compatible pNC1300::Ubi-smGFP destination vector^70^ using LR clonase reactions (Invitrogen). *OsSnRK1a*-RNAi lines used in this study were previously described^18^. All recombination reactions were confirmed by sequencing of the final expression clones.

### Rice transformation

Rice transformation was performed as described previously^70^. In brief, *Agrobacterium tumefaciens* strain EHA105 was used to infect rice callus from cultivar Kitaake for transformation. Transgenic plants were selected on growth media containing hygromycin (40 mg/L) and genotyped by PCR using primers Hyg-F and Hyg-R (Supplementary Table S1) targeting the hygromycin selection marker.

### Rice Pathogen Culture and Inoculation Assays

#### Xanthomonas oryzae pv. oryzae

Bacterial inoculations were performed with *Xoo* strain PXO99^71^ using the standard leaf-clipping method. Before inoculation the pathogen was grown on Sucrose Peptone Agar (SPA) and incubated at 28 °C. For inoculation experiments, single colonies were transferred to liquid SP medium and grown for 24–48 h at 28 °C, until the OD_600_ had reached 0.5. Six-week-old plants were inoculated by clipping the fifth and sixth stage leaves of at least twelve plants with scissors dipped in the *Xoo* suspension. Inoculated plants were kept in a dew chamber (≥92% relative humidity; 28 ± 2 °C) for 24 h and thereafter transferred to greenhouse or growth chamber conditions for disease development. Fourteen days after inoculation, disease severity was assessed by measuring the length of the water-soaked lesions. To measure colony-forming units (CFU), inoculated leaves from 12 plants were grouped in four pools of three plants, ground up thoroughly using mortar and pestle and resuspended in 5 mL water. The leaf suspensions were diluted accordingly and plated on SPA. Plates were incubated at 28 °C in the dark and colonies were counted within 3 days.

#### Pyricularia oryzae

*P*. *oryzae* isolate VT5M1, a field isolate from rice in Vietnam^72^ was grown at 28 °C on half-strength oatmeal agar (Difco, Sparks, USA). Seven-day-old mycelium was flattened onto the medium using a sterile spoon and exposed to blue light (combination of Philips TLD 18 W/08 and Philips TLD 18 W/33) for seven days to induce sporulation. Upon sporulation, conidia were harvested as described by De Vleesschauwer *et al*.^73^ and resuspended in 0.5% gelatin (type B bovine skin, Sigma-Aldrich G-6650). Five-leaf-stage plants were sprayed with spore solution (1 mL per plant) and inoculated plants were kept in a dew chamber (≥92% relative humidity; 28 ± 2 °C) for 18 h and thereafter transferred to growth chamber conditions for disease development. For each line, three trays with six plants each were inoculated. Six days after inoculation, the number of susceptible-type lesions, defined as elliptical to round lesions characterized by a gray center indicative of fungal sporulation, were counted^74^.

#### Cochliobolus miyabeanus

*C*. *miyabeanus* strain Cm988^10^ was grown for sporulation at 28 °C on PDA. Seven-day-old mycelium was flattened onto the medium using a sterile spoon and exposed to blue light for three days under the same conditions mentioned above. Upon sporulation, conidia were harvested exactly as stated in^10^ and resuspended in 0.5% gelatin (type B bovine skin). For inoculation, for each line the two youngest fully developed leaves from 9 individual six-week-old plants were detached, cut into 7 cm segments and immediately placed onto a glass slide in 14.5 × 14.5-cm Petri dishes lined with moist filter paper. Detached leaves were next sprayed until run-off with the spore suspension and incubated under growth chamber conditions (28 °C day/24 °C night, 12 h photoperiod, and 70% relative humidity) for disease development. Disease development was assessed 4 days post inoculation using digital image analysis (APS assess software; Lakhdar Lamari, Winnipeg, Canada) for quantification of symptomatic leaf areas.

#### Rhizoctonia solani

*R*. *solani* isolate 1–16 belonging to anastomosis group AG-1 IA^75^, was maintained on potato dextrose agar (PDA). Detached leaves of 6 individual six-week-old plants were cut into 7 cm segments and immediately placed onto a glass slide in 14.5 × 14.5-cm Petri dishes lined with moist filter paper. For each line, at least 18 leaf segments were inoculated by carefully placing 0.6-cm-diameter agar plugs taken from three-day-old PDA cultures on the adaxial side of the leaves. Five days after incubation under growth chamber conditions (28 °C day/24 °C night, 12 h photoperiod, and 70% relative humidity), diseased leaf area was quantified using APS assess software. Disease ratings were expressed taking into account diseased leaf area using a 1-to-4 disease severity scale: I, less than 25% of leaf area infected; II, between 25–50%; III, 51–75% of leaf area infected; IV, more than 75% of leaf area infected. Disease index (DI) values were calculated according to the following formula: DI = ∑(class × number of plants in class × 100)/total number of plants × 4.

### Hormone Measurements

Collected leaf samples were homogenized using liquid nitrogen; and 100 mg of plant material was extracted for 24 h at −80 °C using 5 mL of the modified Bieleski solvent. After filtration (30 kDa Amicon^®^ Ultra centrifugal filter unit, 30 min, 2900 g, 4 °C) and evaporation (Turbovap^®^, 10 °C, until dryness), the extract was reconstituted with 0.5 mL methanol/water (20:80 v/v) with 0.1% formic acid. Chromatographic separation was performed on a U-HPLC system (Thermo Fisher Scientific) equipped with a Nucleodur C18 column (50 × 2 mm; 1.8 µm d_p_) and using a mobile phase gradient (300 µL/min; solvent A: 0–1 min at 20%, 1–2.5 min from 20 to 45%; 2.5–9 min from 45 to 100%; 9–10 min at 100%; 10–14 min at 20%) consisting of acidified (A) methanol (0.01% formic acid) and (B) water (0.1% formic acid). Mass spectrometric analysis was carried out in selected-ion monitoring (SIM; isolation window 1.0 m/z) mode with a Q Exactive™ Orbitrap mass spectrometer (Thermo Fisher Scientific), operating in negative electrospray ionization mode at a resolution of 70,000 full width at half maximum^67^.

### RNA extraction and Quantitative RT-PCR

Total leaf RNA was extracted using TRIZOL reagent according to the manufacturer’s protocol (Invitrogen) and subsequently treated with Turbo DNase (Ambion) to remove genomic DNA contamination. First-strand cDNA was synthesized from 2 μg of total RNA using Multiscribe reverse transcriptase (Applied Biosystems) and random primers following the manufacturer’s instructions. Quantitative PCR amplifications were conducted in optical 96-well plates with the Mx3005P real-time PCR detection system (Stratagene), using Sybr Green master mix (Fermentas) to monitor dsDNA synthesis. The expression of each gene was assayed in duplicate or triplicate in a total volume of 25 µL including a passive reference dye (ROX) according to the manufacturer’s instructions (Fermentas). The thermal profile used consisted of an initial denaturation step at 95 °C for 10 min, followed by 40 cycles of 95 °C for 15 s, 59 °C for 30 s, and 72 °C for 30 s. To verify amplification of one specific target cDNA, a melting-curve analysis was included according to the thermal profile suggested by the manufacturer (Stratagene). The amount of plant RNA in each sample was normalized using OsACTIN1 (LOC_Os03g50885) as internal control and samples collected from control plants were selected as calibrator. The data were analyzed using Stratagene’s Mx3005P software. Nucleotide sequences of all primers used are listed in Supplementary Table S1.

### Statistical Analyses

All statistical analyses were performed using Graphpad Prism version 6.0 (GraphPad Software, Inc, CA, USA). Data are means ± SD or median and interquartile range based on their distribution (calculated by the D’Agostino-Pearson omnibus K^2^ test). Pearson’s correlation was used. Statistical tests used are indicated in the figure legends or text. A P value of <0.05 was considered statistically significant.

## Electronic supplementary material

Supplementary information
