# Supplementary material for: The energy sensor OsSnRK1a confers broad-spectrum disease resistance in rice
Source: Sci Rep. 2018 Mar 1;8:3864. doi: 10.1038/s41598-018-22101-6 (PMC5832823; doi:10.1038/s41598-018-22101-6)
Supplement: Supplementary file 1 — Supplementary information [file 41598_2018_22101_MOESM1_ESM.pdf]

The energy sensor OsSnRK1a confers broad-spectrum disease resistance in rice

Osvaldo Filipe[1], David De Vleesschauwer[1§] Ashley Haeck[2], Kristof Demeestere[2] and Monica Höfte[1*]

Supplementary information

sequence, LOC_Os05g45420.1

```
MEGAGRDGNPLGGYRIGKTLGIGSFGKVKIAEHILTGHKVAIKILNRRKIKSMEMEEKVK
REIKILRLFMHPHIIRLYEVIDTPADIYVVMEYVKSGELFDYIVEKGRLQEEEARRFFQQ
IISGVEYCHRNMVVHRDLKPENLLLDSKCNVKIADFGLSNVMRDGHFLKTSCGSPNYAAP
EVISGKLYAGPEVDVWSCGVILYALLCGTLPFDDENIPNLFKKIKGGIYTLPSHLSPLAR
DLIPRMLVVDPMKRITIREIREHQWFTVGLPRYLAVPPPDTAQQVKKLDDETLNDVINMG
FDKNQLIESLHKRLQNEATVAYYLLLDNRLRTTSGYLGAEFHESMESSLAQVTPAETPNS
ATDHRQHGHMESPGFGLRHHFAADRKWALGLQSRAHPREIITEVLKALQELNVCWKKIGH
YNMKCRWSPSFPSHESMMHNNHGFGAESAIIETDDSEKSTHTVKFEIQLYKTRDEKYLLD
LQRVSGPQLLFLDLCSAFLTQLRVL*
```

Red: S_TKc = Serine/Threonine protein kinases, catalytic domain
Green: UBA = ubiquitin-associated domain
Blue: KA1 = kinase-associated 1 domain
orange: nucleotide binding region
gray: ATB binding site
Yellow: Activation Loop (A-loop)
Protein length: 505 aa
Nucleotide (CDS): 1518 bp
Mol Wt: 57614.50 dalton
PI: 8.02

Figure S1: OsSnRK1A annotated amino acid sequence.

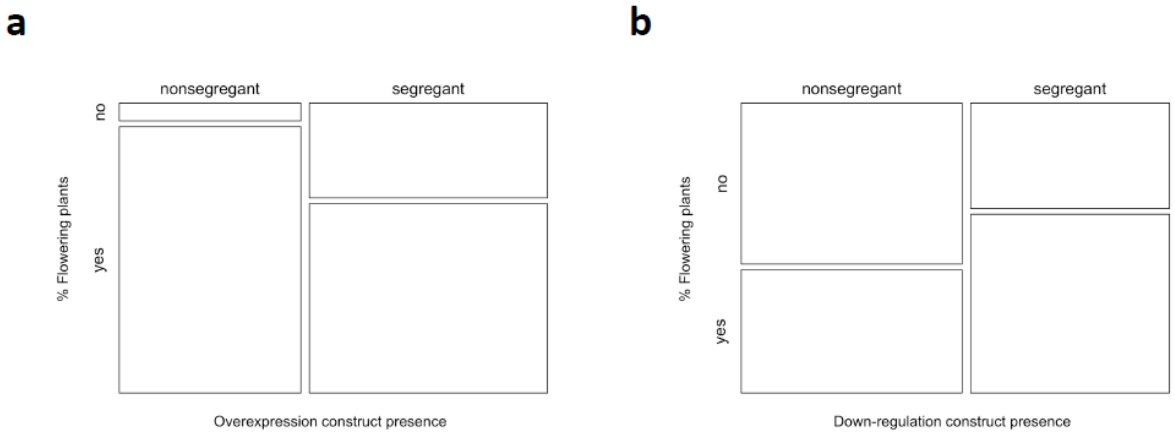

Figure S2. OsSnRK1a negatively affects flowering in rice. (a) *OsSnRK1a* overexpression delays flowering as only 66.67% of transgenic plants displayed flowers compared to 93.75% of azygous controls. (b) *OsSnRK1a* down-regulation positively affects flowering in the Xa21 genetic background as 62.96% of SnRK1 silenced plants flowered compared to 43.33% of Xa21. Plants were grown under greenhouse conditions (a) or under growth chamber conditions (b) and flowering was checked at 50 or 70 days post soil transplantation, respectively.

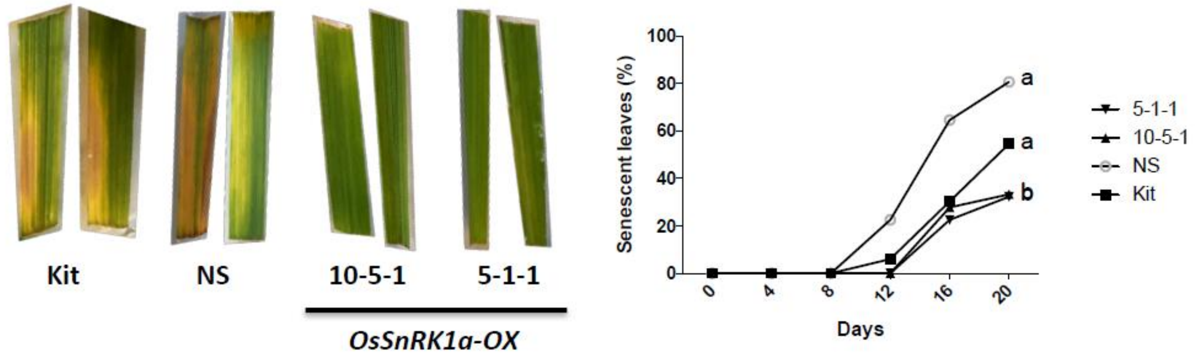

Figure S3. ***OsSnRK1a*** **overexpression delays senescence in rice**. Overexpression of *OsSnRK1a* inhibits senescence-associated yellowing in detached leaf assays compared to wild-type and null segregating plants. The two youngest fully developed leaves from five-leaf stage Kitaake, null segregant and T3 *OsSnRK1a*-OX (10-5-1 and 5-1-1) plants were detached, cut into 5 cm pieces and incubated on sterilized distilled water in closed Petri dishes at room temperature. Pictures were taken 18 days post leaf detachment. Different letters indicate statistically significant differences (Tukey, $n \geq 16$, $\alpha = 0.05$).

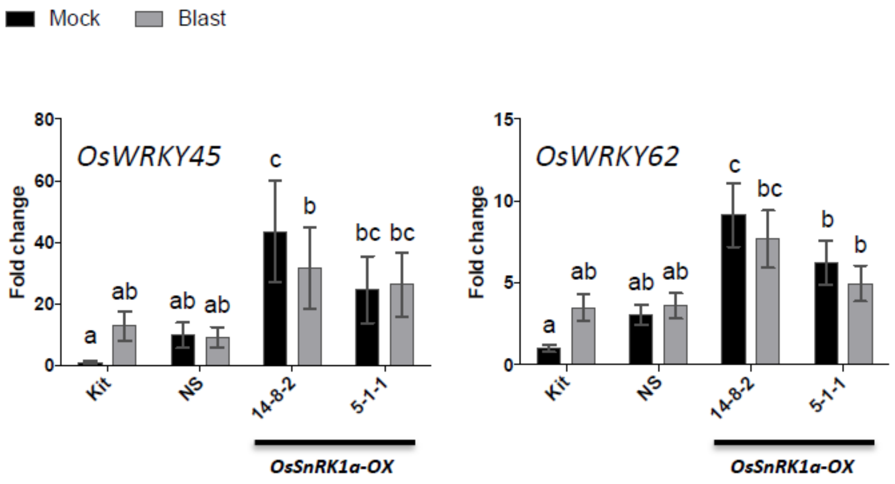

Figure S4. Effect of *OsSnRK1a* overexpression on SA responses 10 days after blast inoculation. *OsSnRK1a* overexpression stimulates expression of SA marker genes (*OsWRKY45* and *OsWRKY62)*. Five-leaf stage Kitaake, null segregant and T3 *OsSnRK1a*-OX (14-8-2 and 5-1-1) plants were treated with a 0.5% gelatin (mock) solution or blast spores solution (VT5M1 at 4.5×10^4 spores/mL). Samples were taken 10 days after treatment. Transcript levels were normalized using actin as an internal reference and expressed relative to the normalized expression levels in mock-inoculated Kitaake plants. Data are means ± SD from three technical replicates of a pooled sample from at least three sibling plants. Different letters indicate statistically significant differences (Tukey, $\alpha = 0.05$).

Supplementary Table S1: Sequences of forward (F) and reverse (R) primers used in genotyping or qRT-PCR

Experiment	Primer name	TIGR Locus ID	Primer sequence (5'-3')
Cloning	OSK1-F	LOC_Os05g45420	CACCATGGAGGGAGCTGGCAGAGATGG
	OSK1-R	LOC_Os05g45420	TTAAAGGACTCTCAGCTGAGTTAGAAAGGCAGAG
Genotyping	Hyg-F		GATGTTGGCGACCTCGT
	Hyg-R		TGTAGGAGGGCGTGGATA
qRT-PCR	Actin-F	LOC_Os03g50885	GCGTGGACAAAGTTTTCAACCG
	Actin-R	LOC_Os03g50885	TCTGGTACCCTCATCAGGCATC
	OsSnRK1-F	LOC_Os05g45420	CGAATCACTTCACAAGAGACTG
	OsSnRK1-R	LOC_Os05g45420	CTGGAGTTACTTGAGCGAGAG
	OsWRKY45-F	LOC_Os05g25770	GGACGCAGCAATCGTCCGGG
	OsWRKY45-R	LOC_Os05g25770	CGGAAGTAGGCCTTTGGGTGC
	OsWRKY62-F	LOC_Os09g25070	AATGCTAGTGGCGACCTACG
	OsWRKY62_R	LOC_Os09g25070	TTGATGGAGATGGAGCACGG
	PR1b-F	LOC_Os01g28450	TATCCAAGCTGGCCATTGCT
	PR1b-R	LOC_Os01g28450	CTCTGGCTGGCGTAGTTCTC
	OsPAL4-F	LOC_Os02g41680	CCCTGCCAATCTGCTGAACTA
	OsPAL4-R	LOC_Os02g41680	GCCGCTATGCAACGAAGAAT
	OsPR10a/PBZ1-F	LOC_Os12g36880	CACCATCTACACCATGAAGC
	OsPR10a/PBZ1-R	LOC_Os12g36880	AGTAGCCATCCACGATGTCC
	JiOsPR10-F	LOC_Os03g18850	CGGACGCTTACAACTAAATCG
	JiOsPR10-R	LOC_Os03g18850	AAACAAAACCATTCTCCGACAG
	OsJAMyb-F	LOC_Os11g45740	GAGGACCAGAGTGCAAAAGC
	OsJAMyb-R	LOC_Os11g45740	CATGGCATCCTTGAACCTCT
	AOS2-F	LOC_Os03g12500	CGTCCAAAGTTTCGGGAGTT
	AOS2-R	LOC_Os03g12500	CTCCATGGCGCCTAGCTAAC

Supplementary Table S2: T1 genotyping and characterization of OsSnRK1a overexpressing transgenic lines used in this study.

Lines (T1)	Plant	Genotyping (Hyg)	Flowering (day 50)	# Tillers	Plant height (cm)	Seed weight (mg)	Xoo lesion length (cm)	
							mean	SD
Kitaake	Kit	negative	yes	7	97.00	10336.40	27.80	2.15
Kitaake	Kit	negative	yes	6	99.50	10759.50	19.50	2.49
5-1	1	positive	yes	3	76.20	3325.60	12.55	1.91
	2	negative	yes	7	80.00	5613.80	18.15	3.95
	3	positive	yes	6	75.00	2974.70	21.73	5.44
	4	positive	yes	2	64.00	1042.90	13.68	3.34
	5	positive	yes	1	49.50	281.60	9.70	6.08
5-3	1	positive	no	2	52.00	271.30	18.43	5.89
	2	positive	yes	3	86.00	5188.00	17.78	4.89
	3	positive	yes	2	85.00	3651.50	21.40	3.21
	4	positive	yes	3	92.00	6140.90	21.53	3.11
	5	positive	no	2	32.00	222.60	16.95	4.03
9-5	1	positive	yes	2	78.00	2095.50	14.50	2.92
	2	positive	yes	2	73.00	1270.20	10.83	4.08
	3	positive	no	3	75.00	1568.80	6.15	1.58
	4	positive	yes	5	91.50	5771.80	17.97	22.59
	5	positive	no	2	69.00	1957.30	11.47	6.00
	6	positive	yes	7	84.20	6244.10	14.78	2.83
10-5	1	positive	no	2	74.00	1352.70	8.97	8.26
	2	positive	yes	5	105.50	4837.70	17.83	5.76
13-2	1	negative	yes	4	87.50	4550.40	20.04	9.62
	2	negative	yes	5	89.00	4088.90	15.13	5.15
	3	negative	yes	5	67.50	3045.20	19.47	4.03
	4	negative	yes	3	74.00	3071.20	19.32	4.37
14-2	1	positive	no	2	53.00	670.30	11.73	1.96
	2	positive	yes	4	87.00	4529.90	22.78	3.19
	3	positive	no	3	82.00	2270.00	18.43	3.72
14-8	1	negative	yes	5	95.50	9299.00	22.60	4.10
	2	positive	yes	3	83.60	4682.70	17.37	3.55
14-10	1	negative	yes	6	93.20	7904.80	28.40	5.50
	2	negative	no	2	49.00	648.30	16.37	5.27
	3	negative	yes	5	81.50	5071.40	21.13	1.37
17-4	1	negative	yes	6	102.00	8010.60	16.04	1.48
	2	negative	yes	3	78.00	3825.30	19.33	3.52
	3	negative	yes	6	90.00	8060.90	22.35	7.78
	4	negative	yes	4	91.00	4061.50	19.98	1.32
	5	negative	yes	5	93.50	5493.70	20.23	2.83

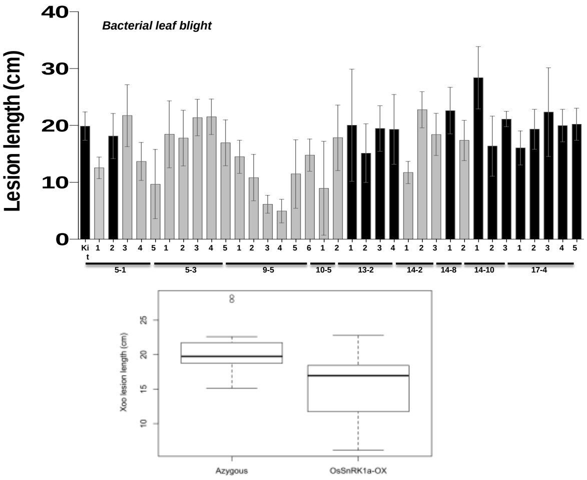

***OsSnRK1a* overexpression positively correlates with resistance to bacterial blight caused by *Xanthomonas oryzae pv.* oryzae (*Xoo*).** Segregating T1 plants grown in greenhouse were genotyped for the presence of the transgene using hygromycin-specific primers. Gray bars represent plants carrying the transgene, black bars indicate null segregants. Eight-week-old plants were inoculated with *Xoo* strain PXO99 using the leaf clipping method and lesion lengths were measured 14 days after inoculation. Data represent means ± SD from at least 3 leaves each. The diagram shows box and whisker (Min to Max) plots of *Xoo* lesion lenghts on transgenes and azygous controls (null segregants and Kitaake wild type). Open circles indicate outliers. Welsh’s t-test revealed significant results with p=0.03754.
